# Devices and tasks involved in the objective assessment of standing dynamic balancing – A systematic literature review

**DOI:** 10.1371/journal.pone.0185188

**Published:** 2017-09-21

**Authors:** Bálint Petró, Alexandra Papachatzopoulou, Rita M Kiss

**Affiliations:** 1 Department of Mechatronics, Optics and Mechanical Engineering Informatics, Budapest University of Technology and Economics, Budapest, Hungary; 2 Department of Mechanical Engineering, University of Thessaly, Volos, Greece; Northwestern University, UNITED STATES

## Abstract

**Background:**

Static balancing assessment is often complemented with dynamic balancing tasks. Numerous dynamic balancing assessment methods have been developed in recent decades with their corresponding balancing devices and tasks.

**Objective:**

The aim of this systematic literature review is to identify and categorize existing objective methods of standing dynamic balancing ability assessment with an emphasis on the balancing devices and tasks being used.

**Data Sources:**

Three major scientific literature databases (Science Direct, Web of Science, PLoS ONE) and additional sources were used.

**Study selection:**

Studies had to use a dynamic balancing device and a task described in detail. Evaluation had to be based on objectively measureable parameters. Functional tests without instrumentation evaluated exclusively by a clinician were excluded. A total of 63 articles were included.

**Data extraction:**

The data extracted during full-text assessment were: author and date; the balancing device with the balancing task and the measured parameters; the health conditions, size, age and sex of participant groups; and follow-up measurements.

**Data synthesis:**

A variety of dynamic balancing assessment devices were identified and categorized as 1) Solid ground, 2) Balance board, 3) Rotating platform, 4) Horizontal translational platform, 5) Treadmill, 6) Computerized Dynamic Posturography, and 7) Other devices. The group discrimination ability of the methods was explored and the conclusions of the studies were briefly summarized.

**Limitations:**

Due to the wide scope of this search, it provides an overview of balancing devices and do not represent the state-of-the-art of any single method.

**Conclusions:**

The identified dynamic balancing assessment methods are offered as a catalogue of candidate methods to complement static assessments used in studies involving postural control.

## Introduction

The assessment of balancing abilities is an integral part of orthopedic and physiotherapeutic evaluation. Balancing as an umbrella term includes the combination of both the control of posture and the control of equilibrium. In this discrimination, postural control encompasses achieving and maintaining a desired body position in any static or dynamic situation. Equilibrium control encompasses maintaining the intersegmental stability of the body in spite of gravitational and inertial forces acting on it [1]. In balancing assessments, a systems approach is used to identify the disordered subcomponents of postural control [2]. Such components of balance control are: maintaining balance, object interaction (‘achieving’ a task) and obstacle negotiation (‘restoring’ balance) [1]. Our study focused on standing dynamic balance in a sense of recovering and/or maintaining standing balance following a sudden perturbation, during a continuous perturbation or under dynamic environmental conditions. Moreover, the field of interest was limited to objective methods of dynamic balancing assessment.

### Balance assessment

The complexity of balancing processes makes it challenging to assess balancing abilities in a concise, holistic approach. Task constraints of balancing assessments in general can be classified as: static body stability (stability to keep the body in a static position), quasi-mobility (dynamic body stability and transfer stability) and mobility (stability during locomotion) [1]. Numerous factors can affect balancing performance. In providing sensory feedback for postural control, there are no corresponding single receptor types but input from numerous sensors are combined [3]. A change in any of these perception methods can change postural control. Cognitive load can also have a major effect on balancing efficiency [4]. The processes of growing up, the effects of young age [5,6] and the deteriorating effects of old age [7] have also been studied extensively.

Balancing abilities can be tested with a functional approach to check for existing balancing problems and to assess the risk of falling. In clinical practice, some of these functional tests are the Berg Balance Scale, the Timed Up-and-Go Test, Functional Ambulation Classification etc. [8]. These tests mainly involve a battery of tasks to be carried out by the participant which are scored by a clinician on a test-specific scale. Such assessment methods provide valuable information on the current motor coordination effectiveness of patients. While functional tests are practical in terms of the low number of required devices and instrumentation, they are inherently subjective as most of them do not use instrumented measurement data in the scoring process. Another method is static posturography, where the participant is standing quietly on a motionless force platform that tracks the COP (center of foot pressure) displacement and calculates postural control measures from COP data. Functional tests and static posturography may not be adequately challenging tests to be completed by athletes or non-athletic healthy individuals. As an example, this may cause failure to discriminate between healthy and asymptomatic individuals in the latter case, while in the former case it may not allow for tracking training progress. To complement these methods, dynamic tests are performed to provide more information on the postural control of the participants by imposing perturbations or placing them in a dynamic environment. For practical reasons and in order to limit the scope of investigation to a certain aspect of balancing, task-specific study methods of dynamic balancing have been developed.

Dynamic balancing is broadly defined as the controlling process taking place under non-static conditions. In order to limit the scope of this study, balancing during standing under dynamic conditions was investigated. Hence, the operational definition of *standing dynamic balancing* is a) the maintenance of standing balance during a continuous perturbation or under dynamic environmental conditions, b) the recovery of standing balance following a sudden perturbation or c) a combination of these. Standing dynamic balance includes both postural control as it aims at maintaining or recovering a balanced standing position and equilibrium control as it must create reactions to destabilizing forces from the perturbation. Note that under this definition, recovery actions can include stepping, i.e. transferring from one standing position to another in a limited number of steps but not walking, i.e. taking continuous steps as in locomotion.

A *dynamic balancing task* or *test* is referred to as an experimental procedure whose aim is to assess the standing dynamic balancing ability of a subject, when one or several types of *external perturbation* or *dynamic conditions* are imposed upon it. Such perturbations can be mechanical stimuli (sudden perturbation or continuous motorized movement of support surface, etc.), sensory stimuli (visual, vestibular or proprioceptive effects) or a combination of such perturbations. Dynamic conditions can be imposed by placing the participant on support surfaces able to move freely or with constraints in order to provide a challenge to maintain postural control. Balancing tasks that fit into this definition potentially activate all components of postural control (‘maintaining’, ‘achieving’ and ‘restoring’). From a task constraint perspective, these tasks fit into the quasi-mobility category, as in keeping the body balanced during movements in one posture or in transferring between standing postures. A *balancing device* is a piece of equipment on which the balancing test can be performed. Various devices, e.g. balancing boards, treadmills, oscillating platforms, force platforms, etc. have been developed for the assessment of balancing abilities. *Ability assessment* refers to the objective method of characterizing balancing. *Measured parameters*, e.g. angle error, COM (center of mass) movement, COP displacements, surface EMG (electromyography), recovery step count, etc. are parameters that are used to evaluate the results of the balancing task. These parameters are usually specific to the study design, thus making it difficult to compare the results of different assessment methods.

### Aim of study

The literature of static balancing assessment methods is well researched and discussed [1,9,10]. However, there seems to be a lack of systematic literature reviews on the objective assessment methods of balancing abilities that correspond to standing dynamic balancing. The aim of this systematic literature review is to identify and categorize existing objective methods of standing dynamic balancing ability assessment with an emphasis on the balancing devices and tasks being used. Synthesis of the collected materials should allow to explore how different methods are able to discriminate between participant groups and which are more promising in research or clinical practice.

## Materials and methods

### Search strategy

#### Identification of materials

This systematic review was carried out according to the PRISMA guidelines [11]. Three electronic databases (Science Direct, Web of Science and PLoS) were searched for publications dated 1997–2017. Key search terms used with Boolean conjunction included: postural control, dynamic balancing, ability, balancing task, perturbation, assessment, human, and additional synonyms of these terms. Search terms were modified according to the required search format of each database. Other sources of materials included comprised reference lists of previously cited articles in our published works on similar topics.

As an example, a full electronic search strategy for the Science Direct database is provided here. In the Advanced search option, the following terms were added with Boolean conjunction to search for in ‘All fields’: (postural control OR postural stability) AND (dynamic balance OR dynamic balancing) AND (ability OR capability) AND (balance task OR balancing task) AND (perturbation) AND (assessment OR evaluation) AND (human OR person OR subjects). The search was refined to journal and book publications. Publication date limits were set to 2007-Present, with the search performed on February 20^th^, 2017. The search of the Science Direct database yielded 577 records. Key search terms were identified and agreed upon by BP and RMK; the electronic search and downloading of results were carried out by BP. Screening, eligibility check of materials and date extraction were carried out by BP and AP.

#### Screening of materials

The identified materials were screened based on title and abstract following the removal of duplicates. Materials of purely theoretical work or with an unrelated topic or aim of study were excluded. Proof of concept articles were not excluded.

#### Eligibility check of materials

To check for eligibility, the reviewers agreed upon a set of inclusion and exclusion criteria (Table 1). Studies had to meet all of the inclusion criteria to be included in the final synthesis. Studies that either met an exclusion criteria or otherwise failed to meet inclusion criteria were excluded. These criteria were set up to provide quality assessment to a certain extent, i.e. the applied methods had to be well communicated and the evaluation of measurement results had to be objective. No additional quality assessment was carried out on the materials included.

**Table 1:** Inclusion and exclusion criteria.

	Inclusion	Exclusion
**Balancing test**	Studies which included *standing dynamic balancing tests* in their experimental procedures.	Studies which only included *static* balancing tests (e.g. quiet standing tests) without any type of external perturbation, dynamic tests other than during standing (e.g. gait analysis or sitting) or other tests not included in our definition of *standing dynamic balancing tests*.
**Description of balancing test**	Studies with detailed descriptions of the balancing test and the experimental process that was followed.	Studies without detailed or incomplete descriptions of the balancing test and the experimental process that was followed.
**Assessment of results**	Studies with objective result assessment based on measurable parameters.	Studies with subjective scoring/assessments of results, not (entirely) based on measurable parameters.

### Data extraction

In accordance to the focus of this review, the final synthesis of the collected material was to extract relevant information on the dynamic balancing ability assessment. The data collected from the articles were: 1) author and date, 2) balancing device, 3) balancing task, 4) measured parameters, 5) health of participants, 6) group size of participants, 7) age and sex of groups, 8) follow-up (the time scale of repeated measurements, if applicable).

## Results

**Fig 1:**
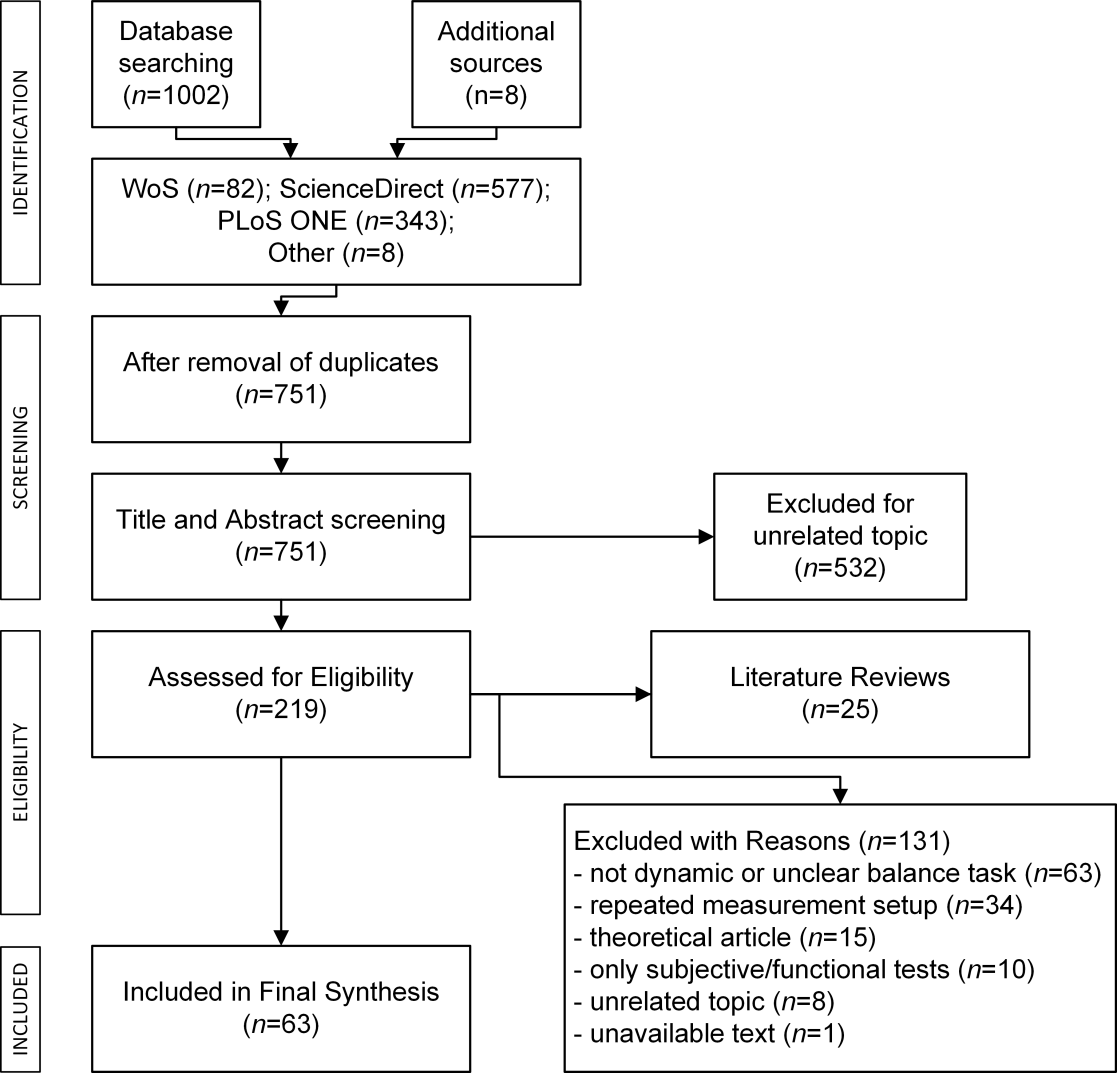
PRISMA flow diagram of systematic review process.

The database search and additional sources yielded 1010 records (Fig 1). After the removal of duplicates and records with missing/unavailable abstracts, 751 records remained. The title and abstract screening excluded 532 records by reason of an unfit topic. The remaining 219 articles underwent full-text eligibility check. Out of the 219 publications, 131 were excluded with reasons, and 25 publications were literature review articles related to postural control and balancing. The review articles found had a different aim and scope from our current study. The number of articles included in the final synthesis was 63 (*n* = 63).

Reasons for exclusion during the full-text eligibility assessment were the following. A large number of studies applied a task outside of the scope of our definition of a standing dynamic balancing task, e.g. quiet standing, and gait analysis. If multiple studies described equivalent balancing devices with the same balancing task and similar instrumentation for evaluation, the earliest publication was included and the others were excluded as ‘repeated measurement setup’. A number of theoretical articles remained at this stage of the screening process, which did not describe a specific measurement setup, and were thus excluded. Some articles operated using a subjective scoring assessment, and a few articles had an irrelevant aim of study, e.g. balancing of bipedal robots.

All extracted data of the materials selected for final synthesis are available in the S1 File. In the case of studies involving multiple measurement setups, only measurements meeting the inclusion criteria were taken into account.

### Overview of dynamic balancing devices

This systematic review aimed at creating a comprehensive catalogue of dynamic balancing assessment methods. A wide variety of balancing devices and corresponding dynamic balancing tasks with an objective evaluation method were found. The testing methods were categorized by the main device being used. The final synthesis identified the main balancing devices as 1) Solid ground, 2) Balance board, 3) Rotating platform, 4) Horizontal translational platform, 5) Treadmill, 6) Computerized Dynamic Posturography, and 7) Other devices. Studies were further differentiated by the specific task. Studies within their respective categories mainly vary in aim, groups of participants, practical application of perturbation, instrumentation and evaluation method.

#### 1. Solid ground

**Fig 2:**
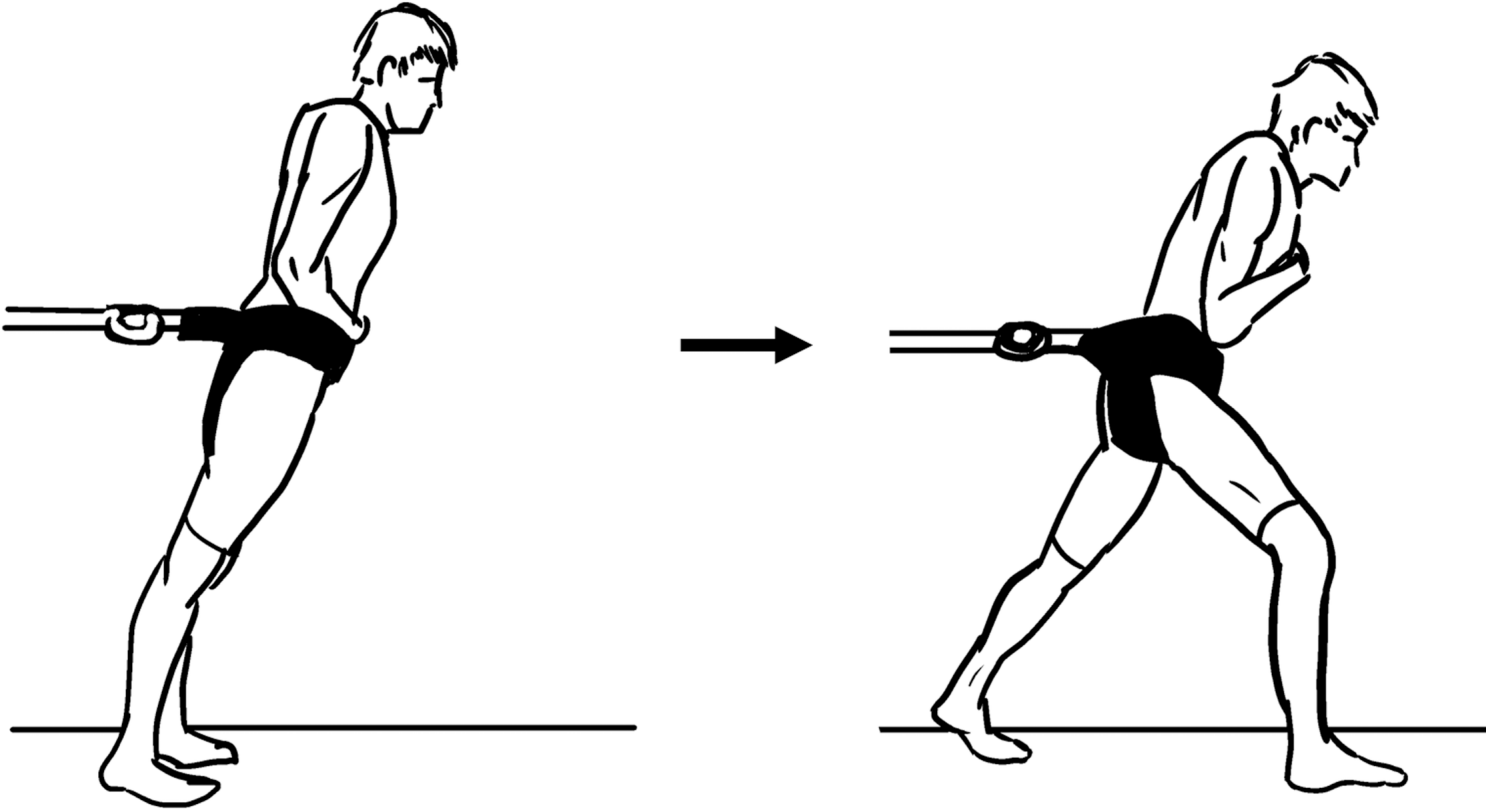
Simulated forward fall method (concept from [13]).

**1.1. Simulated forward fall (release of leaning cable)**: The participant is standing motionlessly, leaning at a forward incline angle to the ground (Fig 2). The leaning is in part or completely supported by a horizontal, taut cable. The maximum angle from which recovery of leaning is possible can be measured [12]. The sudden release of cable tension simulates a forward fall followed by a stepping recovery motion [13–16].

**1.2.** **Pull/push/hit perturbation**

**Fig 3:**
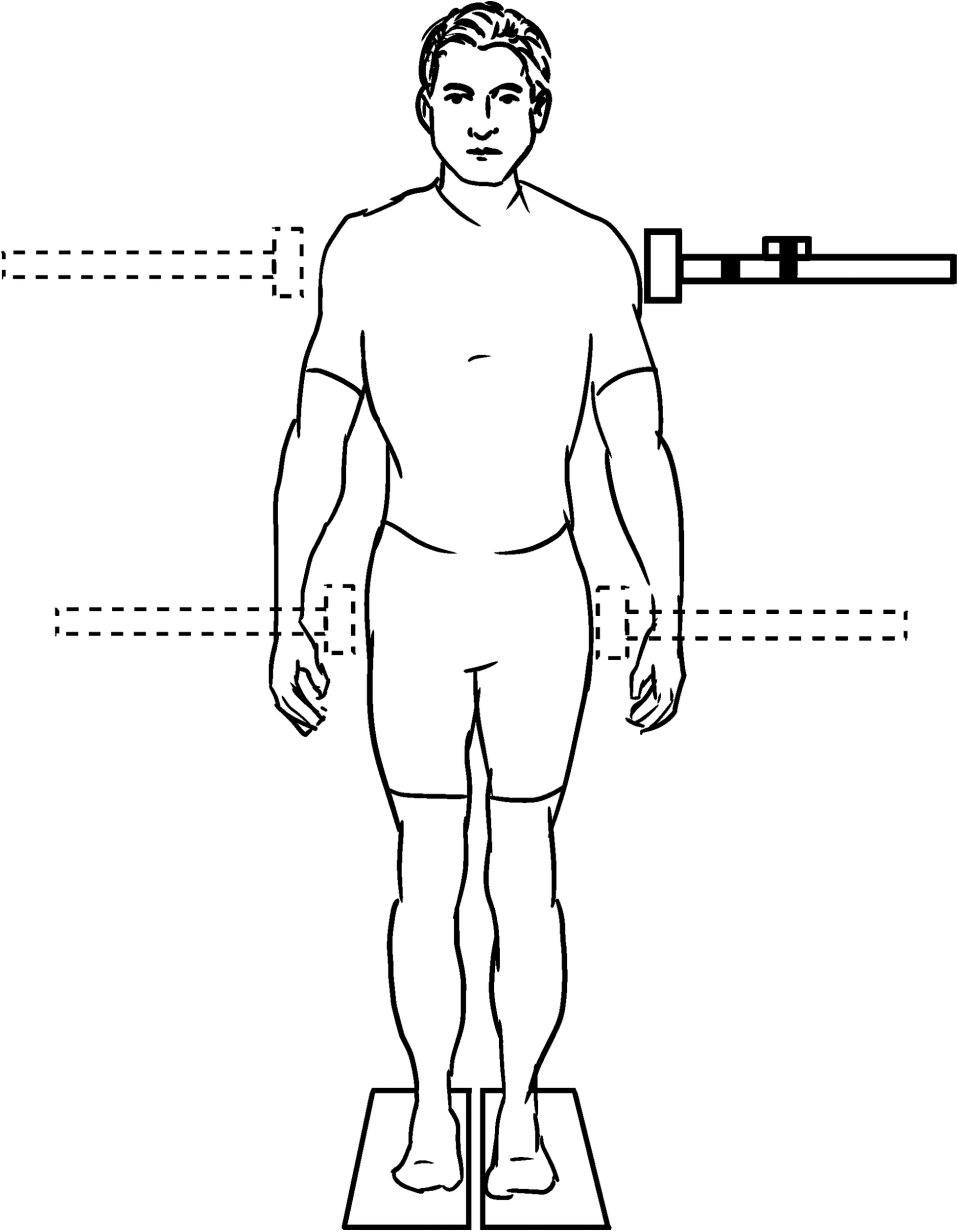
Pull/push perturbations method (concept from [20]).

**1.2.1 Waist pull/push:** The participant is adopting a quiet standing bipedal or single leg stance, with the apparatus in contact at waist level when a sudden pull (push) is applied (Fig 3). The balancing task is the recovery of balance either with or without stepping as instructed specifically. Stepping tasks can be preceded by AP (anterior-posterior) pulls [17] or ML (medio-lateral) pulls [18]. Recovery without stepping can be preceded by AP pulls [19] or AP and ML pulls [20,21]. Pulling perturbation of the waist can be applied on the side of the body, resulting in a rotational perturbation [22].

**1.2.2. Shoulder pull:** A sudden backward (AP) pull is applied to the participant in a quiet standing at shoulder level [23].

**1.2.3. Shoulder hit:** A sudden lateral (ML) push (Fig 3) [20] or hit from a pendulum [24] is delivered to the participant in a quiet standing at shoulder level.

**Fig 4:**
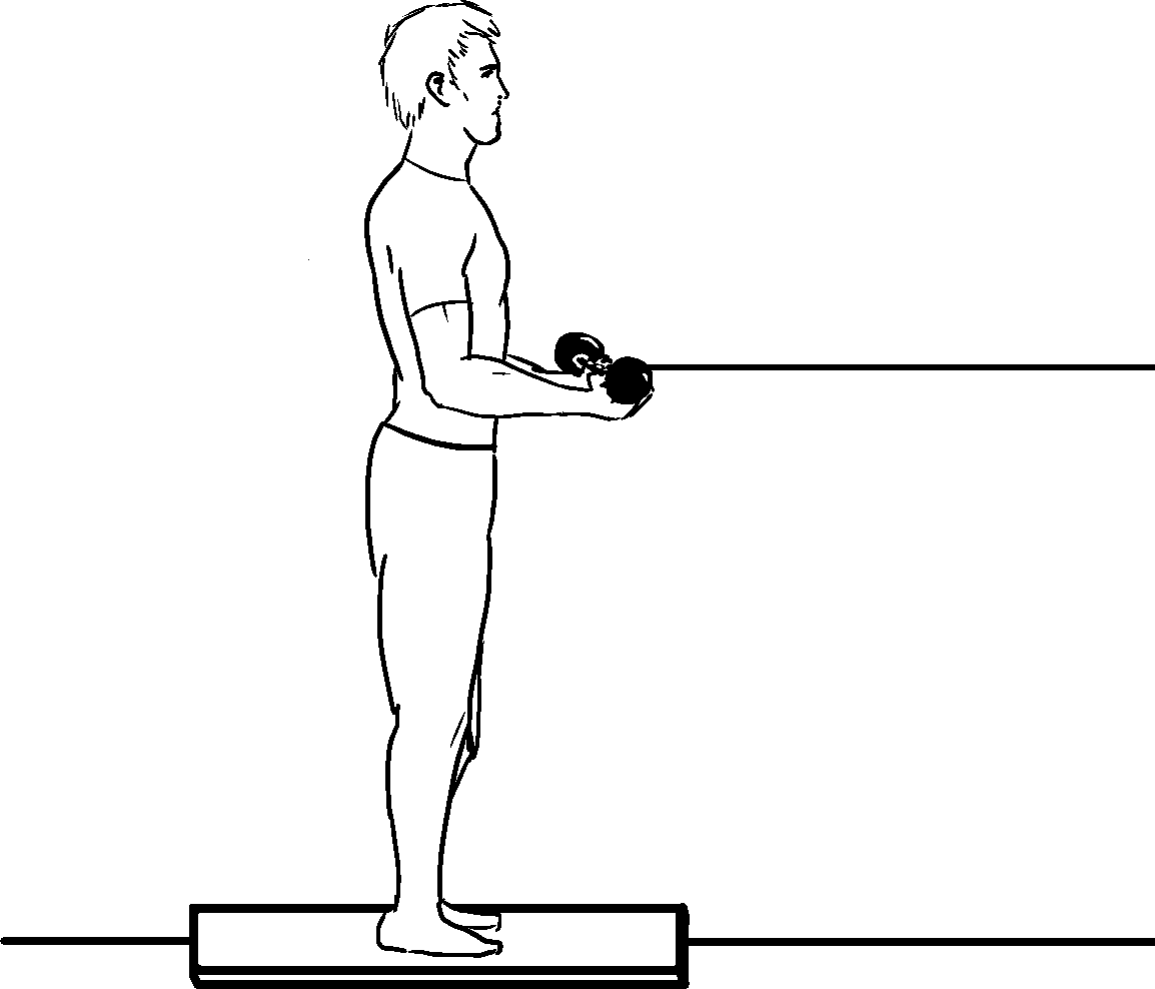
Sudden load on hands method (concept from [27]).

**1.3. Sudden load on hands:** Participants adopting quiet standing on solid or foam surface hold a heavy object in hand (vertical load) that is suddenly released [25]; hold a pan onto which a heavy object is dropped [26]; hold onto a string with a horizontal load that is suddenly released (Fig 4) [27]. The task is to recover standing balance without stepping.

#### 2. Balance board (unperturbed seesaw)

**Fig 5:**
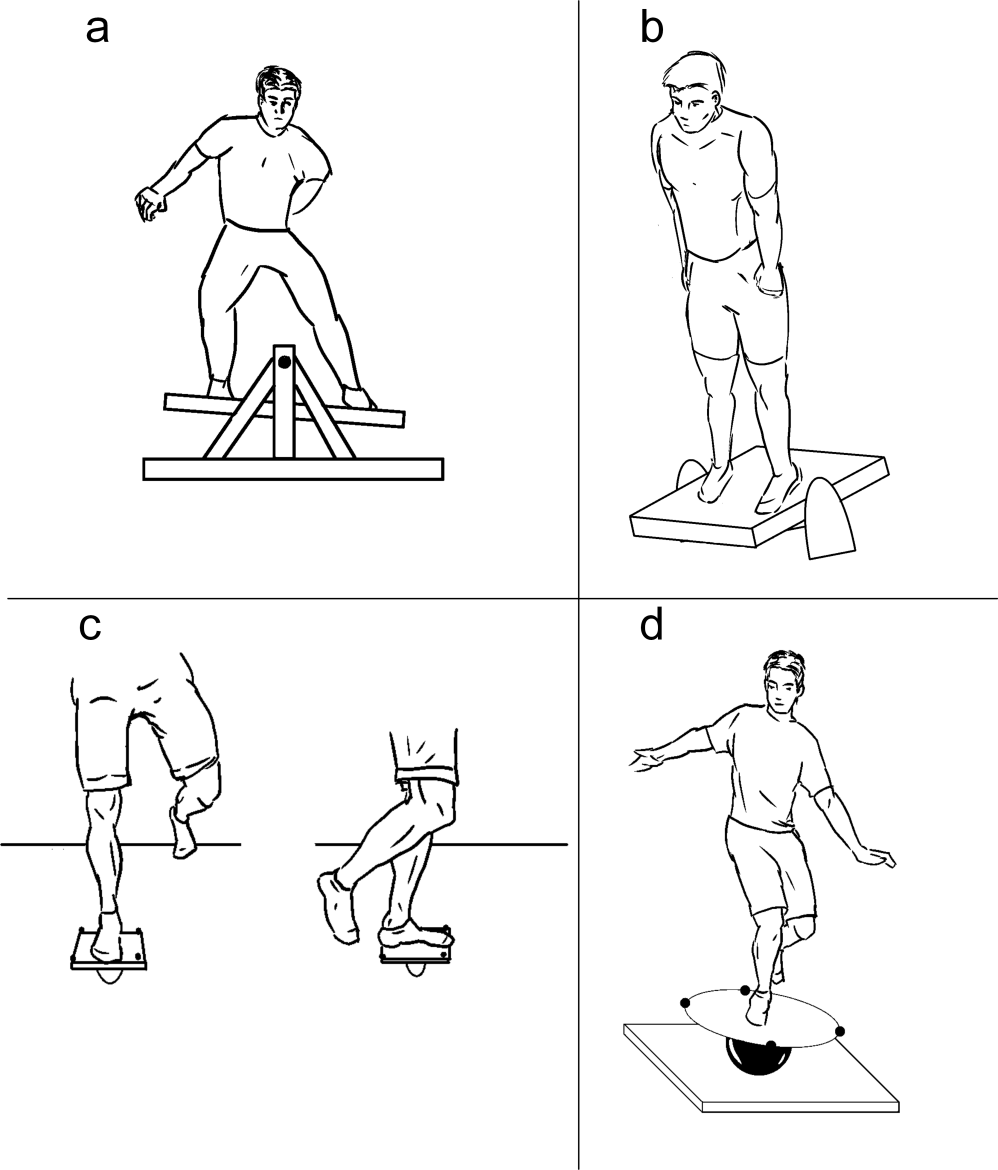
**Balance board methods: a) Sagittal axis (concept from** [29]**), b) Frontal axis (concept from** [32]**), c) Uniaxial (concept from** [36]**), d) Omni-axial.**

**2.1. Sagittal axis balance board (‘stabilometer’):** The participant has to stand on an unstable board that has a sagittal (AP) axis (Fig 5A). The task is to keep the platform horizontal by balancing the weight distribution between legs [28–30].

**2.2. Frontal axis balance board**: The participant has to stand on a board that has a frontal (ML) axis (Fig 5B). The task is to keep the platform horizontal by balancing the weight distribution between the toes and the heels. The measurement can involve changing the degrees of instability by a different support surface [31] or modifying the balance board stiffness [32].

**2.3. Uniaxial balance board**: These devices can provide either a sagittal or frontal axis balance board task depending on the position of the foot on the device (Fig 5C) [33–36].

**2.4. Omni-axial balance board**: The participant is maintaining balance standing on a platform that has a round, hemispheric rocker base (Fig 5D). The platform motion can be tracked optically or by accelerometers, ground reaction forces can be measured by force plate and muscle activation by surface EMG [37,38].

#### 3. Rotating platforms

**Fig 6:**
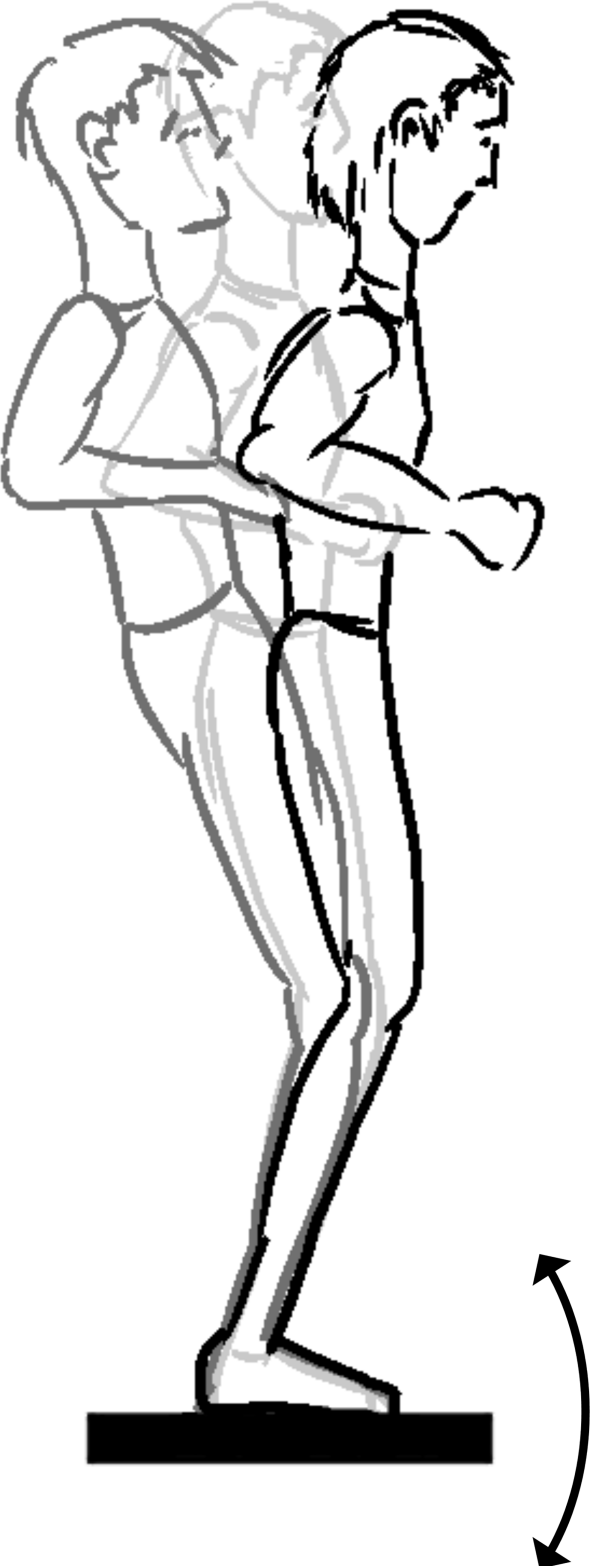
Rotating platform method with sudden or continuous pitch perturbation.

**3.1. Sudden platform rotation perturbation**: The participant is standing on a rigid platform capable of sudden actuated rotation along one or two axes (Fig 6). The perturbation can be a sudden toe-up rotation while the participant is standing freely [39] or being constrained [40]. An ML-AP dual axis platform can deliver rotation perturbation at arbitrary angles [41].

**3.2. Continuous platform rotation perturbation**: Continuous rotations are delivered to the participant standing on a rigid platform (Fig 6). Horizontal rotations (around the axis of the spine) can vary in amplitude and frequency when the body segment motions are analyzed [42]. A single-axis rotational platform can be used to provide continuous pitch perturbation with eyes open and closed conditions to track changes in COP movement [43]. A similar platform can provide pitch or roll rotation perturbation to capture body segment motion [44].

#### 4. Horizontal moving platforms

**Fig 7:**
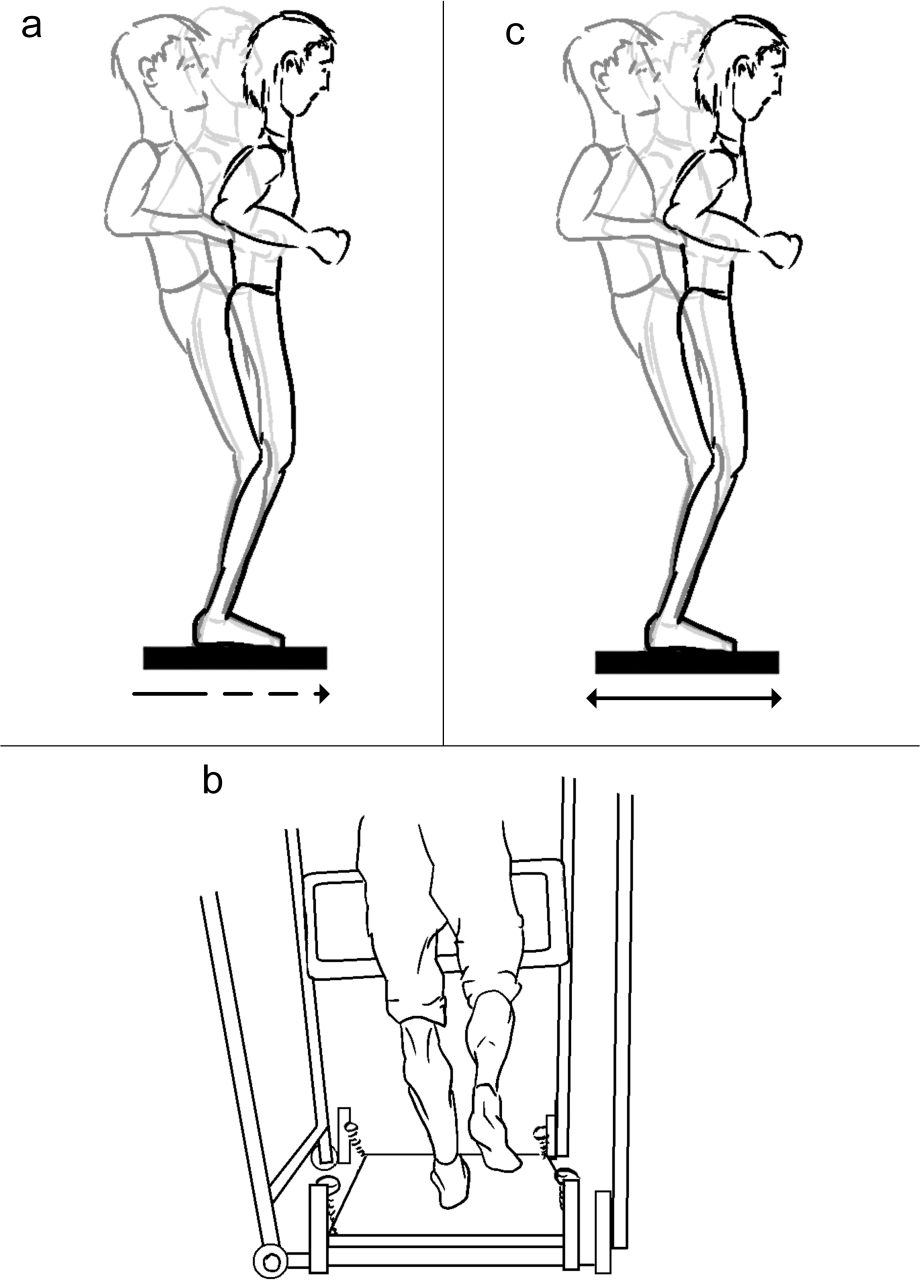
**Horizontal moving platforms: a) Sudden horizontal translation perturbation with controlled stop, b) Sudden horizontal platform perturbation with free oscillation (concept from** [36]**), c) Continuous horizontal oscillating platform perturbation.**

**4.1. Sudden horizontal translation perturbation with controlled stop**: The participant is standing quietly on a horizontal rigid platform when a sudden translational perturbation is delivered (Fig 7A). The platform can have built-in force plates to track COP excursion and the time to stabilization [45,46]. The motion of the platform can be more complex, i.e. translation can change directions [47]. Cognitive tasks can be given to investigate the cognitive contribution to postural control [48], and brain cortex activation can be monitored by applying the perturbation with or without a warning [49].

**4.2. Sudden horizontal platform perturbation with free oscillation**: The participant is adopting a bipedal or single-leg stance on a horizontal rigid platform that is locked outside of its resting position (Fig 7B). The lock is suddenly released to deliver a translational perturbation that is followed by the free oscillation of the platform. The balance recovery actions of the participant act as a damping agent to stop the oscillation. Recovery time, damping factor and EMG activation timing and level can be measured [36,50,51]. A force plate can be fastened onto the platform to allow for COP tracking [52].

**4.3. Continuous horizontal oscillating platform perturbation**: The participant is standing quietly on a horizontal rigid platform and a motorized continuous translational perturbation is applied to the platform in the AP direction (Fig 7C). Body segment movement, EMG and different balancing strategies can be observed [53].The perturbations can scale up and down in frequency [54] and amplitude, and different visual conditions can be applied [55,56]. The frequency changes can be sudden or self-triggered [57].

#### 5. Treadmill

**Fig 8:**
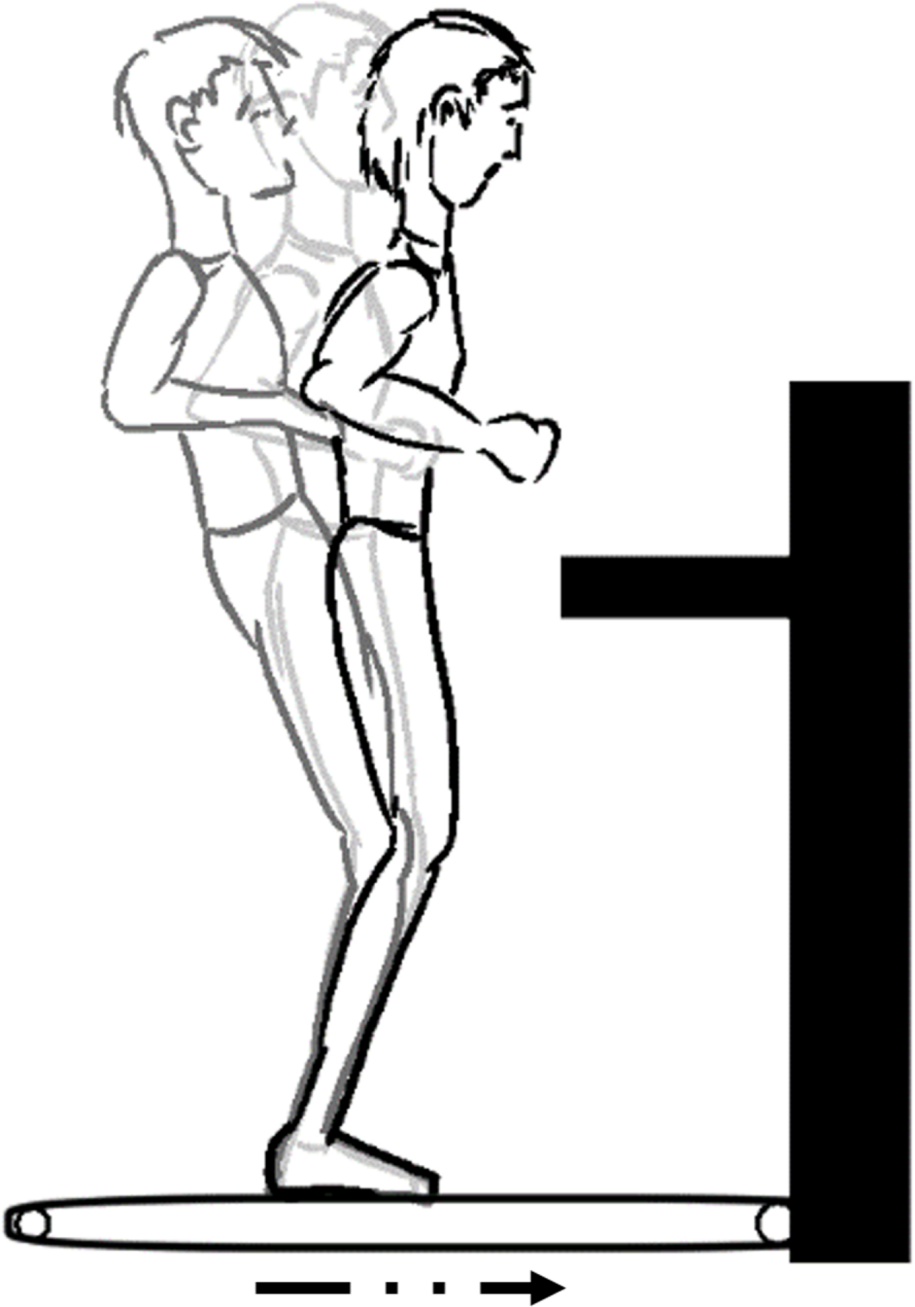
Treadmill method.

**5.1. Sudden horizontal anterior-posterior perturbation:** The participant is quietly standing on a treadmill belt. An anterior [58] or posterior [59] translation perturbation is delivered to elicit a compensatory stepping response (Fig 8). Balance recovery tasks without stepping can also be carried out [60].

#### 6. Computerized Dynamic Posturography

**Fig 9:**
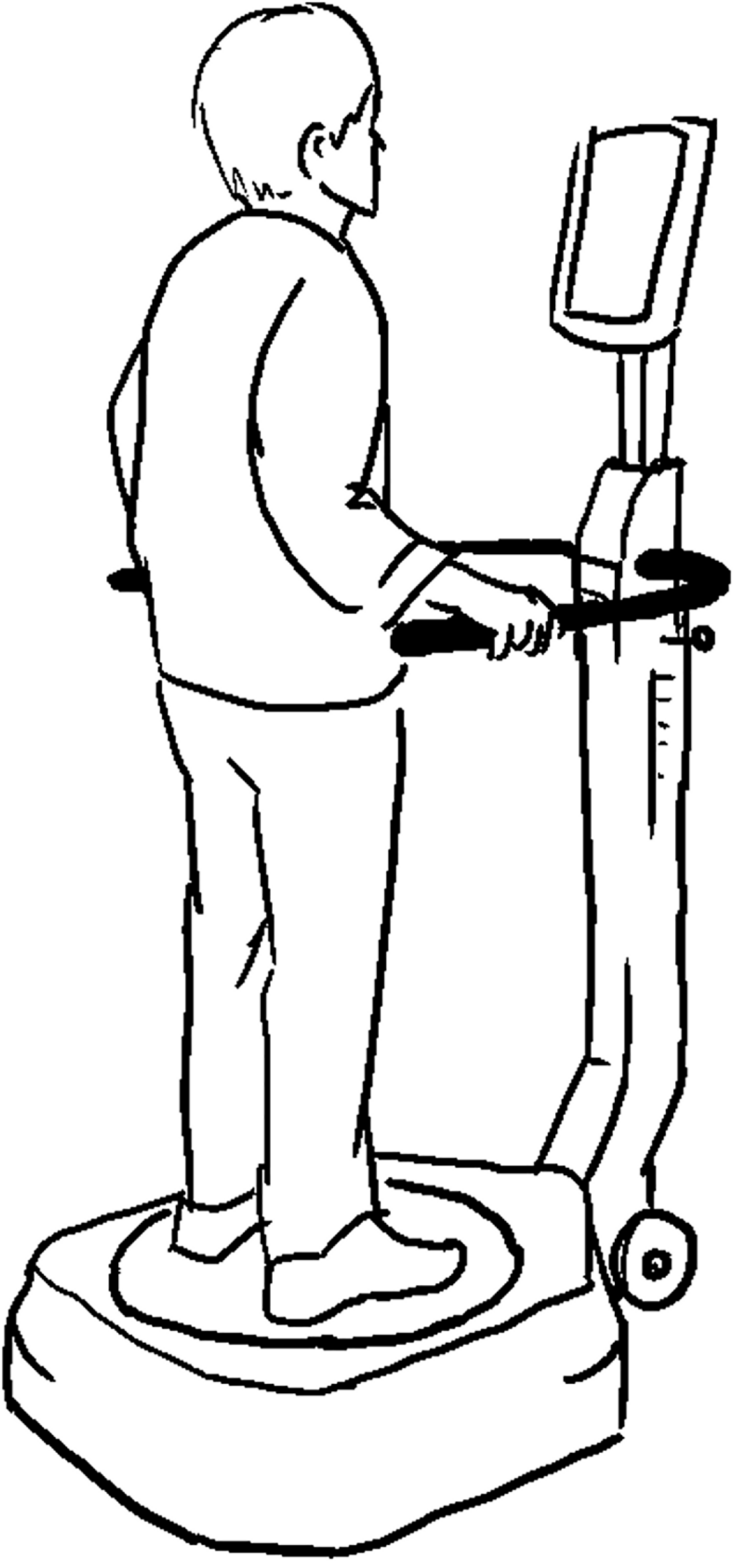
Computerized Dynamic Posturography method.

Computerized Dynamic Posturography is a clinically proven and widely accepted method of assessing balancing abilities. During a CDP (Computerized Dynamic Posturography) test, the participant is standing on a dual force plate support surface (platform) (Fig 9) within a moveable enclosure (visual surroundings). The sensory and motor components in the maintenance of balance can be analyzed under different perturbation conditions (i.e. visual perturbation, platform movement perturbation). Prominent CDP devices and their earliest references identified through our final synthesis were: BIODEX [61]; CAREN [62]; CHATTECX [63]; EQUITEST [64]; FRAMIRAL Multitest Equilibre [65].

#### 7. Other devices

**7.1 Force plate with visual feedback:** A force plate can be used to carry out instructed tasks with visual feedback on a display [66]. The visual feedback can be applied to give a semi-immersive virtual reality balancing task where the participant has to balance a virtual balance board [67].

**7.2 Haptic perturbation:** Visual and haptic sensory inputs are perturbed in quiet standing. COP and EMG data can be collected to track changes in postural control [68].

**7.3 Leg swinging:** The participant is adopting a single-leg stance on a force plate and is instructed to swing the raised leg. EMG monitoring can be applied [69].

**7.4 Objective functional reach tests:** These tests include a functional task with an objectively measureable outcome. Tasks include forward and upward reach with the hands where the reach distance is measured [70] or muscle activation is monitored by EMG [71]. Other reaching tasks are done with the foot, such as the Star Excursion Test [72].

**7.5 Six degrees of freedom platform:** A servo-controlled platform designed to mimic the motion of a ship at sea can be used to deliver continuous perturbation in multiple directions [73].

**7.6 Vibration:** The effect of muscle and tendon vibration perturbation can be analyzed with CDP [74].

## Discussion

### Synthesis of studies

#### Structural framework of dynamic standing balance assessment devices

Balancing devices and tasks can be placed into a proposed structural framework (Table 2). This framework discriminates between methods based on our operating definition of standing dynamic balance, i.e. categorizing tasks into sudden perturbation, continuous perturbation or dynamic condition categories, and on the basis of the main movement constraints imposed upon the participant by the balancing device.

**Table 2:** Structural framework of dynamic standing balance assessment devices.

Movement constraints	Sudden perturbation	Continuous perturbation	Dynamic condition
**No constraints (freely on ground)**	• Simulated forward fall; • Pull/push/hit perturbation; • 1.3. Sudden load on hands	• 7.1 Force plate with visual feedback; • 7.2 Haptic perturbation;	• 7.3 Leg swinging; • 7.4 Objective functional reach tests; • 7.5 Six degrees of freedom platform
**Translational**	• 4.1. Sudden horizontal translation perturbation with controlled stop; • 4.2. Sudden horizontal platform perturbation with free oscillation; • 5. Treadmill*	4.3. Continuous horizontal oscillating platform perturbation	4.2. Sudden horizontal platform perturbation with free oscillation**
**Rotational**	• 3.1. Sudden platform rotation perturbation; • 6. Computerized Dynamic Posturography	• 3.2. Continuous platform rotation perturbation; • 6. Computerized Dynamic Posturography	2. Balance board*

* including all subcategories

**without an applied perturbation

#### Group discrimination and conclusions of studies

The ability of each method to discriminate between different groups of participants was analyzed on the basis of the data extracted from the studies included (S1 File) and are summarized in Table 3. The clinical or theoretical focuses of study conclusions are explored in the following part of this *Discussion* section. Key findings of studies comparing different standing dynamic balancing assessment methods or such methods and static balancing assessment are also communicated here. Remarks on the different study designs and their appropriateness are also made.

**Table 3:** Group discrimination ability of studies involving multiple groups of participants.

	Appropriate to discriminate					
	significantly		to some extent		to a limited or no extent	
Balancing task/device	between groups	study	between groups	study	between groups	study
**Simulated forward fall**	young adult, elderly	[12]	single steppers, multiple steppers	[13]	single steppers, multiple steppers	[14]
	stability training, stability and muscle strength training, control group	[15]				
	PD (Parkinson’s Disease) fallers, PD non-fallers, control	[16]				
**Waist pull/push**	balance impaired elderly, healthy elderly, healthy young	[17]	elderly fallers, elderly non-fallers	[18]		
**Shoulder hit**	elderly fallers, elderly non-fallers, healthy young	[24]				
**Sudden load on hands**	young, early and late middle-aged, physically active and sedentary	[25]	low back pain elderly, healthy control	[27]	low back pain males, low back pain females	[26]
**Sagittal axis balance board**					healthy young adults, learning style groups	[29]
**Uniaxial balance board**	circus-trained children and healthy control	[34]				
	healthy young adults in different training groups	[36]				
**Sudden platform rotation perturbation**			PD, healthy control	[39], [41]		
**Continuous platform rotation perturbation**			female dancers, male judoists, healthy control	[43]		
			PD, healthy control	[44]		
**Sudden horizontal platform**	low back pain, healthy control	[48]				
**Free oscillating platform**	healthy young men, women, healthy elderly men, women	[50]				
	healthy young adults in different training groups	[36]				
**Continuous horizontal oscillating platform**	PD, healthy control	[53]				
	healthy young, healthy elderly	[57]				
**Treadmill, sudden A-P translation**	healthy young adult, middle aged adult, elderly	[58]				
	elderly fallers, non-fallers	[59]				
**CDP**	chronic hemiparesis, healthy control	[62]				
	osteoarthritis, rheumatoid arthritis, healthy control	[63]				
	healthy children, healthy adults	[65]				
**Other:**						
**Leg swinging**	healthy young and elderly	[69]				
**Objective functional reach tests**	healthy young and elderly	[70]	diabetic, diabetic neuropathic, healthy control	[71]		
	healthy athletes with and without specific training	[72]				
**Six degrees of freedom platform**	marine workers, dancers and healthy control	[73]				

The *simulated forward fall method* [12–16] was able to demonstrate significant differences where these differences are highly expected, e.g. between young adults and elderly people, or PD (Parkinson’s Disease) and healthy groups. For example, 90% of PD fallers were correctly classified as such using muscle strength and anterior loss of stability parameters [16]. It also demonstrated some [13] or limited [14] discriminative power between groups of single steppers and multiple steppers, inherently associated with impaired balance performance. Most studies utilizing the simulated forward fall method aimed at reaching clinical conclusions, underlining the importance of exercising dynamic stability control mechanisms [15], which should include balance and agility training in addition to strength training [12] and should address the muscular control of the trunk, the fixed and the stepping limb equally [14].

The *waist pull/push method* [17–22] was applied on a single group in most identified studies [19–22]. The group discrimination can be significant [17] or significant to some extent [18] in the case of pre-diagnosed, evident balance impairment. Leg preference asymmetries were identified in the stepping response, which preferences should be considered in intervention design [18]. Additional steps may also be required in balance impairment and this is due to the failing first step [17]. Most studies reach a theoretical conclusion [19–21]. Ankle stiffness is identified as the first line of defense during this dynamic condition [20] and it is suggested that the AP and ML directional postural control is decoupled by the central nervous system [21]. A study utilizing a cable-pulled belt assessed the validity (*p*<0.001 comparing pre- and post-perturbation) and day-to-day variability (Intraclass Correlation Coefficients 0.81–0.84 for internal, 0.69–0.71 for external rotations), both metrics showing favorable results [22].

In the one study found through our search which specifically utilized a *shoulder pull* [23], the perturbation was applied manually by the same examiner person [23]. It can be recommended to develop a device capable of standardizing such perturbations. The *pendulum hit method* [24] might not be appropriate for some patient populations due to psychological effects, i.e. fear of powerful impact. However, it was used successfully to induce anticipatory postural adjustment in accordance with the aim of study and significantly discriminated between elderly fallers and non-fallers, as well as healthy controls [24].

The *sudden load on hands methods* used either a vertical [25,26] or horizontal [27] release. The vertical design aimed at standardizing the perturbation of clinical vertical push tests, which is a favorable development direction. It successfully discriminated between physically active and sedentary groups [25], but showed only limited differences between males and females with low back pain [26]. Although intended to replace the vertical push test, this sudden perturbation clearly elicits reactive balance control actions, which is not the explicit aim of the corresponding clinical test. The horizontal release study proved less effective at discriminating between groups of low back pain patients and healthy controls [27]. However, this horizontal design resembles situations encountered in the everyday lives of patients, which makes it a favorable candidate method of further studies.

The *balance board* is a common way of providing unstable, dynamic conditions for balancing tests. The included balance board studies that focused on a single (either sagittal or frontal) axis of motion investigated only single groups. *Sagittal axis balance board* studies [28–30] had distinct clinical conclusions: a concurrent verbal task can improve balancing performance on a board [29]; significant learning occurs during six consecutive days of training on a board [28]; fatigue affects static and dynamic stability differently, static balancing decreasing significantly more [30]. Utilizing *frontal axis balance boards* [31,32], a proof of concept article provides insight into postural control changes with changing board stiffness [32]. Another study found that the ankle strategy is most prominent in this task [31], similarly to quiet standing.

*Uniaxial balance boards* [33–36] have been deployed in studies in recent years to assess its appropriateness both in testing and training. The earliest study found rapid adaptation which infers with the testing ability of the seesaw [33]. Indeed, this method effectively discriminated between circus-trained children and healthy controls [34]. However, the difference decreased in eyes closed condition. The transitional learning effects between a uniaxial balance board and a sudden horizontal translation platform with free oscillation were assessed [36], concluding that no cross-training occurred. It also follows that clinicians should identify and train exactly the tasks that need improvement [36]. An advantage of this device is that it can be used to provide both frontal and sagittal axis rotation conditions based on foot orientation, where significantly different muscle activation can be observed [35].

*Omni-axial balance boards* [37,38] as a research device garnered interest only in recent years. Comparing the results of quiet standing and omni-axial balance board tests, no significant correlations between similar parameters were found [38]. This proves that such boards demand a biomechanical control strategy different from quiet standing, i.e. they require more than the ankle strategy, which is favorable in eliciting postural responses of interest. However, the balancing task is learnt rapidly and such skills are retained as tested in a one week follow-up study [37].

The *sudden rotating platform* [39–41] was used with participants suffering from PD and it demonstrated some level of discriminatory power compared to healthy controls [39,41]. One study [39] examined the learning effects associated with this balancing task. They found that first trial reactions can significantly discriminate between PD patients and controls, and learning affects the results. Although learning is slower for patients, the difference between groups eventually disappears [39]. The other study of PD patients found that a reduced flexibility of the trunk and pelvis contributes the most to balance deficits [41]. An early study on a healthy group focused on reaching theoretical conclusions on the ankle strategy [40].

*Continuous rotating platforms* [42–44] also discriminated between groups only to some extent. Using eyes open and eyes closed measurements, one study concluded that the impaired proprioception of PD patients can be compensated by visual dependence and this can be defined as an adaptive strategy [44]. Another study compared high-level judoists, ballet dancers and healthy controls, concluding that the balance strategies and techniques adopted by judoists should be considered for incorporation into rehabilitation programs [43]. One study utilized horizontal rotations along the vertical axis and assessed the compensatory balance reactions in healthy subjects [42]. Notably, only one other study utilized a similarly directed rotation, but with sudden perturbations [22]. Therefore, it is suggested that further studies be carried out using the horizontal continuous rotation method.

A platform actuated with *sudden horizontal translation perturbation with controlled stop* [45–49] was able to significantly discriminate between low back pain and healthy control groups [48]. This method also provides a well-defined motion trajectory which can be adjusted to fit populations with impaired functions as demonstrated on post-stroke patients [45,46]. The controlled motion of the platform makes it a suitable candidate for sensitive neurological measurements to be carried out during balancing, such as monitoring cortical activity using near-infrared spectroscopy [49].

The *sudden perturbation free oscillating platform* [50–52] showed excellent group discriminatory powers both between groups of healthy young men, women, healthy elderly men, women [50] and significantly detected the effects of balance training [51]. The inter- and intraday reliability of the free oscillating platform proved favorable and at least 12 trials can be carried out without significant learning effects [52]. Another advantage of this method is that the perturbation is standardized, and following the perturbation the platform provides an unstable dynamic condition (Table 2). Such platforms are widely used in orthopedics for diagnostics and follow-up measurements.

A *continuously oscillating horizontal platform* [53–57] was able to discriminate significantly between PD patients and healthy controls [53] as well as healthy young and elderly groups [57]. The method detected abnormal temporal features in balancing strategy adaptation in PD patients [53]. Self-triggered changes in the perturbation also leads to different strategies in the elderly [57]. A measurement protocol for clinicians is offered in [55]. It is noteworthy that all studies used only AP perturbation [53–57] while the same device could be used to deliver ML perturbation.

A *treadmill* [58–60] was utilized to deliver a sudden anterior-posterior perturbation only in recent years. The method was able to significantly discriminate between healthy groups of young, middle-aged and elderly adults [58]. Furthermore, the treadmill method showed significant differences between elderly fallers and non-fallers, even when clinical tests failed to discriminate [59]. One study investigated the reliability of this method using computerized dynamic posturography and found good reliability (Intraclass Correlation Coefficients >0.6) and moderate correlation (r>0.5) of the results [60]. These results indicate that the use of a treadmill in clinical practice merits further development. It is worthwhile to explore the interchangeability of actuated horizontal moving platforms (categories 4.1 and 4.3 in the overview) and treadmills. A treadmill could be used to deliver horizontal AP and ML continuous sinusoidal perturbation in a future study. It is also to be noted that only sudden AP perturbation was used with the treadmill as the balancing device (category 5.1). The same measurement setup could be used to apply a sudden ML perturbation adopting a single-leg or bipedal stance.

In the identified studies, *CDP methods* [61–65] were utilized to provide a standardized measure of balancing abilities. CDP methods are well-equipped to discriminate between groups, as demonstrated in studies on chronic hemiparesis and control groups [62], as well as patients suffering from osteoarthritis, rheumatoid arthritis and healthy controls [63], and between healthy adults and children [65]. All included studies reached clinical conclusions, e.g. the contribution of each ankle to balancing can be quantified in the case of hemiparesis [62]. CDP can be a complementing method as part of a battery of balance assessment tests [63]. One study found that in the case of otherwise healthy participants suffering from a temporary balance impairment, dynamic head tilts may improve the diagnostic sensitivity of CDP [64]. These computerized systems can provide balance training as well, of which one significantly improved balance measures following a 4-week training [61]. The standardized nature of CDP also makes it a suitable method for long-term follow-up studies, such as following the improvement of children’s balance control through ageing [65].

A brief remark on studies that utilized an *uncategorized assessment method* [66–73] is given in the following section. Efforts were made to develop the force plate method with visual feedback to provide an objective, on-field assessment of impairment, i.e. driving under the influence [66]. The approach of another study utilizing the force plate with a semi-immersive virtual reality feedback is noteworthy, since in this setup less motion is required from the participant. This can provide a valuable diagnostic tool in the case of patients in a fragile health state [67]. The use of a haptic perturbation, combined with different visual conditions is also of interest because the nature of such perturbations are purely sensory. As such, the sensorimotor integration, attention shifting and other components of postural control can be studied separately [68]. Providing tendon vibrations can also be regarded as delivering a continuous sensory perturbation [74]. The study utilizing leg swinging is of interest since it combines a task that is functional to some extent and also a self-imposed continuous perturbation. They found weaker muscle synergy coupling and a lack of coordination in older adults and successfully discriminated between them and healthy young adults [69]. Studies with objective functional reach tests significantly discriminated between healthy young and elderly groups [70] and to some extent also between diabetic, diabetic neuropathic, and healthy control groups [71]. This method can also be used to track the effects of different training and exercise programs over time [72]. One of the most versatile balance assessment devices is the six-degrees-of-freedom servo-controlled platform that mimics sea motion. The method excellently discriminated between marines, dancers and healthy controls; groups that had different experiences under such unstable conditions. They concluded that short-term adaptation was dependent on the nature of previous long term experiences [73].

### Limitations of this study

Since this study focused on collecting different methods of assessment and proof-of-concept articles were also included, the bias of publishing only positive results is thus minimized. However, regarding the group discriminatory power of measurement methods (Table 2), it is possible that studies that failed to discriminate between groups were less likely to be published. The different measurement methods did not allow for meta-analysis or additional analysis (e.g., subgroup analyses). This systematic review was not registered and thus no review protocols are available online or otherwise. Other limitations can arise from the fact that only three major scientific databases were used in the search for materials. While this limitation can be addressed by including more of the reference lists of eligible articles, the quantity of included materials would make the results unpresentable. To limit the number of included studies, only the earliest appearance of a respective method was included, thus the conclusions derived might not reflect the state-of-the-art status of any single balance assessment method. It is suggested that subsequent reviews should be carried out with a smaller scope, i.e. focusing on a single balancing device. In this case, it would also be favorable to collect the parameters used in measurement evaluation which allows for a meta-analysis of the results.

## Conclusion

The complexity of both postural control and maintenance of balance makes it challenging to assess balancing abilities in a concise, holistic approach. It seemed more feasible to study dynamic balancing using well-defined, task-specific methods. This systematic literature review set out to identify and categorize existing objective measurement methods in the study of standing dynamic balancing abilities. The final synthesis identified the main balancing devices as 1) Solid ground, 2) Balance board, 3) Rotating platform, 4) Horizontal translational platform, 5) Treadmill, 6) Computerized Dynamic Posturography, and 7) Other devices. Only one of these methods, i.e. Computerized Dynamic Posturography is standardized and widely accepted as a reference method. Non-standard balance assessment methods have their own corresponding numerical parameters of evaluation and there is little overlap in these parameters between different methods of assessment. However, the various methods offer different motion constraints and perturbation types, out of which researchers and clinicians can choose the most appropriate one for their work. The identified dynamic balancing assessment methods were categorized and placed into a proposed framework of standing dynamic balancing assessment methods. The ability of these methods to discriminate between groups was explored through the results of collected studies and remarks were made on their conclusions. These results are offered as a catalogue of candidate methods to complement static balancing assessments used in studies involving postural control.

## Supporting information

S1 File Tables of all extracted data from articles used in final synthesis. Records are categorized corresponding to the balance device categories presented in the *Results* section of the paper.
(DOCX)

S2 File PRISMA checklist. (DOC)
