# Supplementary material for: Devices and tasks involved in the objective assessment of standing dynamic balancing – A systematic literature review
Source: PLoS One. 2017 Sep 21;12(9):e0185188. doi: 10.1371/journal.pone.0185188 (PMC5608356; doi:10.1371/journal.pone.0185188)
Supplement: S1 File — Records are categorized corresponding to the balance device categories presented in the Results section of the paper. (DOCX) [file pone.0185188.s001.docx]

Abbreviations and symbols used in this file:

EMG – electromyography

MoCap – motion capture system (optical)

COP – center of (foot) pressure

COM – center of mass

COG – center of gravity

AP – anterior-posterior

ML – medio-lateral

* – information not available

- – not applicable

Age: mean age ± standard deviation in years

Sex: M/F: Male/Female, number of participants

| **1. Solid Ground** | | | | | | | | | |
| --- | --- | --- | --- | --- | --- | --- | --- | --- | --- |
| **1.1. Simulated forward fall** | | | | | | | | | |
| **Author and Date** | **Balancing device and apparatus** | | **Balancing Task** | **Measured Parameters** | **Health** | **Group size** | **Age, Sex** | **Follow-up** | |
| [Mackey & Robinovitch, 2006] | Horizontal leaning cable; Force plate; EMG | | Simulated forward fall: recovering w/ and w/o release of cable | Maximum initial lean angle; Ankle torque; Reaction time; EMG. | G1: young  G2: elderly | G1: n=25  G2: n=25 | G1: age: 25±4  M/F: 0/25  G2: age: 78±7  M/F: 0/25 | - | |
| [Arampatzis, Peper, & Bierbaum, 2011] | Horizontal leaning cable; Force plate; | | Simulated forward fall | Ground reaction forces | G1: stability training  G2: stability and muscle training  G3: control | G1: n=13  G2: n=13  G3: n=12 | G1: age: 66.6±1.8  M/F: 4/9  G2: age: 68.3±2.7  M/F: 2/11  G3: age: 68.3±3.3  M/F: 5/7 | 14 weeks | |
| [Cronin, Barrett, Lichtwark, Mills, & Carty, 2013] | Horizontal leaning cable; Force plate; EMG | | Simulated forward fall, recovery w/ stepping | Ground reaction forces; EMG | G1: single steppers  G2: multiple steppers | G1: n=36  G2: n=30 | G1: age: 69±3  M/F: 25/11  G2: age: 70±3  M/F: 13/17 | - | |
| [Graham, Carty, Lloyd, Lichtwark, & Barrett, 2014] | Horizontal leaning cable; Force plate; MoCap | | Simulated forward fall, recovery w/ stepping | Ground reaction forces; Full-body MoCap; Calculated muscle forces | G1: older adults  G2: younger adults | G1: n=10  G2: n=5 | G1: age: 70±3.9  M/F: 10/0  G2: age: 34±2.6  M/F: 10/0 | - | |
| [Moreno Catalá, Woitalla, & Arampatzis, 2015] | Horizontal leaning cable; Force plate; MoCap | | Simulated forward fall, recovery w/ stepping | AP limit of stability; Full-body MoCap; COM | G1: healthy control  G2: PD non-fallers  G3: PD fallers | G1: n=14  G2: n=13  G3: n=12 | G1: age: 47±5  M/F: */*  G2: age: 47±6  M/F: */*  G3: age: 49±4  M/F: */* | - | |
| **1.2. Pull/push/hit perturbation** | | | | | | | | | |
| **1.2.1 Waist pull/push** | | | | | | | | | |
| **Author and Date** | | **Balancing device** | **Balancing Task** | **Measured Parameters** | **Health** | **Group size** | **Age, Sex** | | **Follow-up** |
| [Schulz, Ashton-Miller, & Alexander, 2005] | | Pull cable attached to belt; Force plate | Balance recovery w/ steps after randomized AP waist pulls at quiet standing | Foot position and velocity; COM velocity; Step count; Step distance and duration | G1: balance impaired elderly  G2: healthy elderly  G2: healthy young | G1: n=15  G2: n=12  G2: n=13 | G1: age: 76  M/F: 0/15  G2: age: 71  M/F: 0/12  G2: age: 23  M/F: 0/13 | | - |
| [Young, Whitall, Bair, & Rogers, 2013] | | Pull cable attached to belt; Force plate | Balance recovery w/ steps after ML waist pulls at quiet standing | Step count; Leg preference; Balance Tolerance Limit | G1: healthy fallers G2: healthy non fallers | G1: n=13 G2: n=23 | G1: age: 73.4±1.1  M/F: */*  G2: age: 74.6±1.6  M/F: */* | | - |
| [Pollock, Ivanova, Hunt, & Garland, 2015] | | Pull cable attached to belt; Force plate; EMG | Maintenance of balance after AP waist pulls at quiet standing | COM velocity; COP velocity; EMG; Ankle torque | chronic stroke | n=9 | age: 68.11±7.67  M/F: 7/2 | | - |
| [Rietdyk, Patla, Winter, Ishac, & Little, 1999] | | Push perturbation device; Force plate; MoCap | Maintenance of balance after experimenter-delivered ML push at waist or shoulder | COP/COM displacement; Body segment displacements; Trunk and shank angles | healthy | n=10 | age: 26.0±4.2  M/F: */* | | - |
| [Matjacic, Voigt, Popovic, & Sinkjaer, 2001] | | Waist perturbation apparatus; Force plate | Maintenance of balance after perturbation in 8 directions | Ground reaction forces; Body segment angles; Calculated Net Joint Torque | healthy | n=8 | age: 31±6.7  M/F: 8/0 | | - |
| [Shultz et al., 2000] | | Pull cable attached to sides of belt; Force plate; EMG | Maintenance of balance after rotational waist perturbation | EMG; Muscle reaction time | healthy athletes | n=64 | age: 20.0±1.2  M/F: 32/32 | | - |
| **1.2.2 Shoulder pull** | | | | | | | | | |
| **Author and Date** | | **Balancing device** | **Balancing Task** | **Measured Parameters** | **Health** | **Group size** | **Age, Sex** | | **Follow-up** |
| [Caudron et al., 2014] | | Experimenter-delivered backward pull; MoCap | Maintenance of balance after experimenter-delivered backward (AP) pull at shoulder | Full-body MoCap | Parkinson’s Disease | n=17 | age: 61.9 ± 8.2  M/F: 10/7 | | - |
| **1.2.3 Shoulder hit** | | | | | | | | | |
| **Author and Date** | | **Balancing device** | **Balancing Task** | **Measured Parameters** | **Health** | **Group size** | **Age, Sex** | | **Follow-up** |
| [Claudino, dos Santos, & Santos, 2013] | | Pendulum w/ weight at the end; Force plate; EMG | Maintenance of balance after pendulum hit (ML) at shoulder level | EMG; COP | G1: old non-fallers  G2: old fallers  G3: young control | G1: n=20  G2: n=20  G3: n=20 | G1: age: 72.89 ± 6.30  M/F: */*  G2: age: 75.60 ± 5.98  M/F: */*  G3: age: 23.85 ± 2.70 years;  M/F: */* | | - |
| **1.3 Sudden load on hands** | | | | | | | | | |
| **Author and Date** | | **Balancing device** | **Balancing Task** | **Measured Parameters** | **Health** | **Group size** | **Age, Sex** | | **Follow-up** |
| [Sung & Park, 2009] | | Force plate; Hand-held pan | Maintenance of standing stability during perturbation due to load drop | Pain levels, Ground reaction forces; Response time; | G1: Low back pain males  G2: Low back pain females | G1: n=18  G2: n=18 | G1: age: 49.9±8.0  M/F: 18/0  G2: age: 51.7±7.5  M/F: 0/18 | | **-** |
| [Zemková, Štefániková, & Muyor, 2016] | | Force plate; Hand-held bar | Maintenance of standing stability during perturbation due to random release of bar | COP sway | G1: young physically active  G2: young sedentary  G3: early middle-aged physically active  G4: early middle-aged sedentary  G5: late middle-aged physically active  G6: late middle-aged sedentary | G1: n=49  G2: n=50  G3: n=36  G4: n=38  G5: n=39  G6: n=39 | G1: age: 21.8  M/F: 24/25  G2: age: 21.6  M/F: 29/21  G3: age: 35.2  M/F: 17/19  G4: age: 36.9  M/F: 16/22  G5: age: 58.2  M/F: 15/24  G6: age: 53.8  M/F: 12/27 | | **-** |
| [P.-Y. Lee et al., 2016] | | Force plate; Pulling rope | Maintenance of standing stability during perturbation due to release of pull rope | Number of falls; COP motion, velocities and sway | G1: healthy control  G2: Low Back Pain | G1:n=26  G2:n=30 | G1: age: 66.23±4.53  M/F: 15/11  G1: age: 64.57±5.71  M/F: 12/18 | | **-** |

| **2. Balance board (unperturbed seesaw)** | | | | | | | |
| --- | --- | --- | --- | --- | --- | --- | --- |
| **2.1. Sagittal axis balance board (‘stabilometer’)** | | | | | | | |
| **Author and Date** | **Balancing device** | **Balancing Task** | **Measured Parameters** | **Health** | **Group size** | **Age, Sex** | **Follow-up** |
| [Mégrot & Bardy, 2006] | Sagittal (AP) axis balance board; MoCap | Keeping the platform horizontal by weight distribution between legs. | Body segment variations | healthy | n=6 | age: *  M/F: */* | 6 consecutive days |
| [Orrell, Eves, & Masters, 2006] | Sagittal (AP) axis balance board | Keeping the platform horizontal by weight distribution between legs. | Platform angle error | * | n=42 | age: 20.29  M/F: */* | - |
| [Marcolin et al., 2016] | Sagittal (AP) axis balance board; Force plate | Keeping the platform horizontal by weight distribution between legs. | Time of successful balancing; COP; Platform acceleration | healthy athletes | n=20 | age: 46.5±8.3  M/F: 20/0 | - |
| **2.2. Frontal axis balance board** | | | | | | | |
| **Author and Date** | **Balancing device** | **Balancing Task** | **Measured Parameters** | **Health** | **Group size** | **Age, Sex** | **Follow-up** |
| [Almeida, Carvalho, & Talis, 2006] | Frontal (ML) axis balance board; EMG; MoCap | Keeping the platform horizontal by weight distribution between toes and heels. | EMG; Joint kinematics | * | n=6 | age: 24.5  M/F: 3/3 | - |
| [Chagdes, Rietdyk, Jeffrey, Howard, & Raman, 2013] | Frontal (ML) axis balance board; Force plate | Keeping the platform horizontal by weight distribution between toes and heels. | COP; COM | **PROOF OF CONCEPT ARTICLE** | - | - | - |
| **2.3. Uniaxial balance board** | | | | | | | |
| **Author and Date** | **Balancing device** | **Balancing Task** | **Measured Parameters** | **Health** | **Group size** | **Age, Sex** | **Follow-up** |
| [Rougier, 2012] | (1) Single axis balance board; Force plate  (2) Static posturography force plate | (1) Keeping the platform horizontal with AP and ML axis, eyes closed  (2) Quiet standing, eyes closed | COP; | healthy | n=32 | age: 21-26*  M/F: 25/7 | - |
| [Sahli et al., 2013] | (1) Single axis balance board; Force plate  (2) Static posturography force plate | (1) Keeping the platform horizontal with AP and ML axis, eyes open/closed  (2) Quiet standing, eyes open/closed | (1) COP trajectory  (2) COP trajectory | G1: circus trained children G2: healthy children | G1: n=12 G2: n=12 | G1: age: 5-6*  M/F: 7/5  G2: age: 5-6*  M/F: */* | - |
| [De Ridder, Willems, De Mits, Vanrenterghem, & Roosen, 2014] | Single axis balance board; EMG | Keeping the platform horizontal with AP, ML, diagonal etc. axis | EMG | healthy | n=69 | age: 21.8±1.7  M/F: 31/38 | - |
| [Giboin, Gruber, & Kramer, 2015] | (1) Single axis balance board;  (2) PosturoMed | (1) Keeping the platform horizontal with AP and ML axis  (2) Regaining balance after sudden perturbation | (1) Platform angle; Time at equilibrium (2) Platform displacement; Time to equilibrium | G1: PosturoMed training group  G2: Balance Board training group  G3: Control | G1: n=14  G2: n=14  G3: n=12 | G1: age: 26±5  M/F: */*  G2: age: 24±3  M/F: */*  G3: age: 23±3  M/F: */* | 2 weeks |
| **2.4. Omni-axial balance board** | | | | | | | |
| **Author and Date** | **Balancing device** | **Balancing Task** | **Measured Parameters** | **Health** | **Group size** | **Age, Sex** | **Follow-up** |
| [Valle, Casabona, Cavallaro, Castorina, & Cioni, 2015] | Balance board with hemispheric base; Force plate | Keeping the platform horizontal | Angular position of platform; COP | healthy | n=10 | age: 24.8±3.3  M/F: 10/10 | 1 week |
| [Silva, Oliveira, Mrachacz-Kersting, Laessoe, & Kersting, 2016] | (1) Balance board with hemispheric base; Force plate under the base of platform; EMG; Optical markers  (2) Static posturography force plate | (1) Keeping the platform horizontal  (2) Quiet standing with eyes open | (1) EMG; Body segment velocities  (2) EMG; COP; | healthy | n=17 | age: 28±4  M/F: 17/0 | - |

| **3. Rotating platform** | | | | | | | |
| --- | --- | --- | --- | --- | --- | --- | --- |
| **3.1. Sudden platform rotation perturbation** | | | | | | | |
| **Author and Date** | **Balancing device** | **Balancing Task** | **Measured Parameters** | **Health** | **Group size** | **Age, Sex** | **Follow-up** |
| [Nanhoe-Mahabier et al., 2012] | Servo-controlled dual-axis platform; MoCap; | Maintenance of balance following successive sudden toe-up pitch rotation perturbation | COM; | G1: Parkinson’s Disease  G2: healthy control | G1: n=8  G2: n=8 | G1: age: 57.5±8.9  M/F: 7/1  G2: age: 53.4±7.0  M/F: 7/1 | - |
| [Mihelj, Matjačić, & Bajd, 2000] | Mechanical rotating frame for lower body with actuated base; MoCap; EMG | Maintenance of balance following successive sudden toe-up pitch rotation perturbation w/ lower body constraints | EMG; Joint angles | healthy | n=8 | age: 23±4  M/F: 8/0 | - |
| [Visser et al., 2008] | Servo-controlled dual-axis platform; MoCap; EMG | Maintenance of balance following sudden ankle rotations in arbitrary directions | Body segment motion; EMG; | G1: Parkinson’s Disease  G2: healthy control | G1: n=14  G2: n=18 | G1: age: 50.2±8.3  M/F: 10/4  G2: age: 50.8±8.1  M/F: 9/8 | - |
| **3.2. Continuous platform rotation perturbation** | | | | | | | |
| **Author and Date** | **Balancing device** | **Balancing Task** | **Measured Parameters** | **Health** | **Group size** | **Age, Sex** | **Follow-up** |
| [Cappa et al., 2008] | Servo-controlled vertical axis platform; MoCap | Maintenance of balance during continuous horizontal rotation | Body segment angles; | healthy | n=10 | age: 30±9  M/F: 3/7 | - |
| [Perrin, Deviterne, Hugel, & Perrot, 2002] | (1) Servo-controlled single-axis platform; Force plate  (2) Static posturography with force plate | (1) Quiet standing with eyes open/closed  (2) Maintenance of balance during continuous pitch rotation | (1) COP;  (2) COP; | G1: female dancers  G2: male judoists  G3: healthy control | G1: n=14  G2: n=17  G3: n=42 | G1: age: 22.1±4.5  M/F: 0/14  G2: age: 24.8±4.5  M/F: 17/0  G3: age: 23.9±4.2  M/F: 21/21 | - |
| [Vaugoyeau, Viel, Assaiante, Amblard, & Azulay, 2007] | Servo-controlled single-axis platform; MoCap | Maintenance of balance during continuous pitch or roll rotation | Body segment angles; | G1: Parkinson’s Disease  G2: healthy control | G1: n=11  G2: n=10 | G1: age: 60.1±6.4  M/F: */*  G2: age: 58.1±6.2  M/F: */* | - |

| **4. Horizontal moving platform** | | | | | | | |
| --- | --- | --- | --- | --- | --- | --- | --- |
| **4.1. Sudden horizontal translational perturbation with controlled stop** | | | | | | | |
| **Author and Date** | **Balancing device** | **Balancing Task** | **Measured Parameters** | **Health** | **Group size** | **Age, Sex** | **Follow-up** |
| [Vearrier, Langan, Shumway-Cook, & Woollacott, 2005] | Servo-controlled translation platform; Force plate | Recovery of balance w/o stepping after sudden backward translation perturbation | COP; Recovery time; Stepping threshold | Post-stroke (medically stable) | n=10 | age: 59±18  M/F: 6/4 | 3 months |
| [de Kam, Kamphuis, Weerdesteyn, & Geurts, 2017] | Servo-controlled translation platform; Force plate | Recovery of balance w/o stepping after sudden translation perturbation in 4 directions | Ground reaction forces; Stepping threshold | Post-stroke | n=14 | age: 62±9  M/F: 11/3 | - |
| [Welch & Ting, 2014] | Servo-controlled translation platform; Force plate; EMG; MoCap | Recovery of balance w/o stepping after complex sudden translation | EMG; COP; COM; | healthy | n=15 | age: 22.5±3.2  M/F: 7/8 | - |
| [Etemadi, Salavati, Arab, & Ghanavati, 2016] | Servo-controlled translation platform; Force plate | Recovery of balance w/o stepping after sudden backward or forward translation perturbation with concurrent cognitive task | COP; Reaction time; | G1: Low back pain  G2: healthy control | G1: n=20  G2: n=20 | G1: age: 31.68±8.63  M/F: 10/10  G2: age: 30.95±8.08  M/F: 11/9 | - |
| [Mihara, Miyai, Hatakenaka, Kubota, & Sakoda, 2008] | Pneumatic translation platform; Functional Near Infrared Spectroscopy | Recovery of balance w/o stepping after sudden backward or forward translation perturbation w/ and w/o audible warning | Platform motion; Cortical activity; | healthy | n=15 | age: 29.4±6.7  M/F: 9/6 | - |
|  | | | | | | | |
| **4.2. Sudden horizontal translation perturbation with free oscillation** | | | | | | | |
| **Author and Date** | **Balancing device** | **Balancing Task** | **Measured Parameters** | **Health** | **Group size** | **Age, Sex** | **Follow-up** |
| [Kiss, 2011] | Free oscillating platform (PosturoMed); Ultrasound tracking | Recovery of balance after sudden unidirectional (ML) translation perturbation | Platform motion; Time of recovery; Damping factor | G1: healthy young men  G2: healthy young women  G3: healthy elderly men  G4: healthy elderly women | G1: n=10  G2: n=10  G3: n=8  G4: n=12 | G1: age: 22.7±3.5  M/F: 10/0  G2: age: 27.5±6.3  M/F: 0/10  G3: age: 71.4±2.4  M/F: 8/0  G4: age: 70.4±3.1  M/F: 0/12 | 7 weeks |
| [Pfusterschmied et al., 2013] | Free oscillating platform (PosturoMed); MoCap; EMG | Recovery of balance after sudden unidirectional (ML) translation perturbation | EMG; Body segment angles; Platform motion | healthy | n=24 | age: 24±0.7  M/F: 12/12 | - |
| [Schmidt, Germano, & Milani, 2015] | Free oscillating platform (PosturoMed); Force plate | Recovery of balance after sudden unidirectional (ML and AP) translation perturbation | COP; Platform motion | healthy | n=30 | age: 24.3±3.2  M/F: 15/15 | 2 days |
| [Giboin et al., 2015] – cited before, see section 2.3 |  |  |  |  |  |  |  |

| **4.3. Continuous horizontal translation perturbation with oscillation** | | | | | | | |
| --- | --- | --- | --- | --- | --- | --- | --- |
| **Author and Date** | **Balancing device** | **Balancing Task** | **Measured Parameters** | **Health** | **Group size** | **Age, Sex** | **Follow-up** |
| [De Nunzio, Nardone, & Schieppati, 2007] | Servo-controlled translation platform; EMG; MoCap | Maintenance of balance during sinusoidal AP perturbation w/ different visual conditions | COP; EMG; Body segment motion | G1: Parkinson’s Disease  G2: healthy control | G1: n=19  G2: n=13 | G1: age: 71.7±7.6  M/F: 10/9  G2: age: 69.8±6.3  M/F: */* |  |
| [Ko, Challis, & Newell, 2014] | Servo-controlled translation platform; Force plate; MoCap | Maintenance of balance during sinusoidal AP perturbation | COP; COM; Joint angles | healthy | n=10 | age: 30.6±3.3  M/F: 10/0 | - |
| [Buchanan & Horak, 2003] | Servo-controlled translation platform; Force plate; | Maintenance of balance during sinusoidal AP perturbation w/ different visual conditions | COP; | healthy | n=6 | age: 34.5±5.6  M/F: 2/4 | - |
| [Schmid, Bottaro, Sozzi, & Schieppati, 2011] | Servo-controlled translation platform; Sensorized insoles; EMG; MoCap | Maintenance of balance during sinusoidal AP perturbation w/ different visual conditions | COP; COM; EMG | healthy | n=10 | age: 27±5.4  M/F: 2/8 | - |
| [Bugnariu & Sveistrup, 2006] | Servo-controlled translation platform; Force plate; | Maintenance of balance during sinusoidal AP perturbation w/ self-triggered and sudden frequency change | COP; | G1: healthy young  G2: healthy elderly | G1: n=8  G2: n=8 | G1: age: 22±2  M/F: 4/4  G2: age: 70±5  M/F: 4/4 | - |

| **5. Treadmill** | | | | | | | |
| --- | --- | --- | --- | --- | --- | --- | --- |
| **5.1. Sudden horizontal anterior-posterior perturbation** | | | | | | | |
| **Author and Date** | **Balancing device** | **Balancing Task** | **Measured Parameters** | **Health** | **Group size** | **Age, Sex** | **Follow-up** |
| [Crenshaw & Grabiner, 2014] | Controlled treadmill; MoCap | Balance recovery w/o steps after sudden anterior translation perturbation | Margin of stability; Treadmill belt velocity; | G1: healthy young adult  G2: healthy middle-aged adult  G3: healthy elderly | G1: n=13  G2: n=11  G3: n=11 | G1: age: 31.1±0.8  M/F: 6/7  G2: age: 57.6±2.5  M/F: 6/5  G3: age: 73.8±5.3  M/F: 6/5 | - |
| [Honeycutt, Nevisipour, & Grabiner, 2016] | Controlled treadmill; MoCap | Balance recovery w/ steps after sudden anterior or posterior translation perturbation | Margin of stability; Body segment angles | G1: elderly fallers  G2: elderly non-fallers | G1: n=10  G2: n=7 | G1: age: 61.7±3.4  M/F: 7/3  G2: age: 57.7±2.5  M/F: 7/0 | - |
| [Yuntao et al., 2017] | (1) Controlled treadmill; Force plate  (2) EquiTest | (1) Balance recovery w/o steps after sudden anterior or posterior translation perturbation  (2) CDP | (1) COG  (2) CDP score | healthy | n=10 | age: *  M/F: */* | - |

| **6. Computerized Dynamic Posturography** | | | | | | | |
| --- | --- | --- | --- | --- | --- | --- | --- |
| **Author and Date** | **Balancing device** | **Balancing Task** | **Measured Parameters** | **Health** | **Group size** | **Age, Sex** | **Follow-up** |
| [Akhbari, Ebrahimi Takamjani, Salavati, & Ali Sanjari, 2007] | Biodex CDP device | Standardized CDP | Standardized CDP | healthy | G1: n=15 | G1: age: 24.3±4.1  M/F: 7/8 | 4 weeks |
| [van Asseldonk et al., 2006] | CAREN CDP device | Standardized CDP | Standardized CDP | G1: chronic hemi paretic  G2: healthy control | G1: n=8  G2: n=6 | G1: age: 59.9±8.3  M/F: 8/7  G2: age: 61.4±3.4  M/F: 6/0 | - |
| [Hill et al., 2013] | Chattecx CDP device | Standardized CDP | Standardized CDP | G1: osteoarthritis  G2: rheumatoid arthritis  G3: healthy control | G1: n=17  G2: n=17 G3: n=17 | G1: age: 66.9±9.8  M/F: 0/17  G2: age: 66.3±9.4  M/F: 0/17  G3: age: 66.3±10.1  M/F: 0/17 | - |
| [Paloski et al., 2006] | EquiTest CDP device | Standardized CDP | Standardized CDP | healthy | n=12 | age: 22-50  M/F: 6/6 | - |
| [Gouleme, Ezane, Wiener-Vacher, & Bucci, 2014] | Framiral CDP device | Standardized CDP | Standardized CDP | G1: healthy children  G2: healthy adults | G1: n=46  G2: n=13 | G1: age: 9.3±3  M/F: 20/26  G2: age: 25±3  M/F: */* | - |

| **7. Other devices** | | | | | | | |
| --- | --- | --- | --- | --- | --- | --- | --- |
| **Author and Date** | **Balancing device** | **Balancing Task** | **Measured Parameters** | **Health** | **Group size** | **Age, Sex** | **Follow-up** |
| [Rossi & Pascolo, 2015] | Force plate with visual display feedback | Controlled COP movement; Reaction to visual stimuli | COP control accuracy; Choice reaction time | healthy | n=10 | age: 30.1±11.2  M/F: 5/5 | 6 months |
| [Basso Moro et al., 2014] | Virtual reality headset; Force plate; Functional Near Infrared Spectroscopy | Maintenance of balance on a virtual ML-axis balance board | COM trajectory; Prefrontal cortex activity | healthy | n=16 | age: 29.0±4.8  M/F: 16/0 | - |
| [Sozzi, Do, Monti, & Schieppati, 2012] | Haptic device; Force plate; EMG; Video | Maintenance of balance during visual-haptic perturbation | COP; EMG; Finger force; Eyelid movement | healthy | n=10 | age: 25.6±5.23  M/F: 5/5 | - |
| [Hatzitaki, Amiridis, & Arabatzi, 2005] | Force plate; Video | Maintenance of balance in single leg stance during leg swinging | Leg position; COP; COG | G1: healthy elderly  G2: healthy young | G1: n=11  G2: n=9 | G1: age: 70.1±4.3  M/F: */*  G2: age: 20.1±2.4  M/F: */* |  |
| [Row & Cavanagh, 2007] | Force plate; Sliding hand plate | Maintenance of balance during forward/upward reaching | Reach distance; Loss of stability; Balancing confidence | G1: healthy young  G2: healthy elderly | G1: n=21  G2: n=31 | G1: age: 24.1±3.0  M/F: 10/11  G2: age: 82.4±2.7  M/F: 17/14 | - |
| [Maranesi et al., 2016] | Force plate; EMG; MoCap | Functional React Test | EMG; Reach distance; Joint angles | G1: healthy control  G2: diabetic  G3: diabetic neuropathy | G1: n=10  G2: n=10  G3: n=10 | G1: age: 73.5±5.1  M/F: */*  G2: age: 72.6±4.1  M/F: */*  G3: age: 72.1±5.0  M/F: */* | - |
| [Rasool & George, 2007] | Solid ground | Star Excursion Balance Test | Reach distance with feet | G1: healthy athletes with specific training  G2: healthy athletes control | G1: n=16  G2: n=14 | G1: age: 21.5±5.1  M/F: 16/0  G2: age: 21.0±4.2  M/F: 14/0 | 2, 4 weeks |
| [Duncan, Ingram, Mansfield, Byrne, & McIlroy, 2016] | Servo-controlled 6 degrees of freedom platform; Force plate; Camera | Recovery of balance w/ stepping during simulated ship board motion | COP; Times of changing support; Step count | G1: healthy marines  G2: healthy dancers  G3: healthy control | G1: n=14  G2: n=13  G3: n=12 | G1: age: 30.9±5.3  M/F: */*  G2: age: 27.1±4.2  M/F: */*  G3: age: 26.8±4.7  M/F: */* | - |
| [Dettmer, Pourmoghaddam, O’Connor, & Layne, 2013] | EquiTest CDP device; Tendon vibration device | Quiet standing during Achilles tendon vibration perturbation | Standardized CDP | healthy | n=18 | age: 24.1±4.2  M/F: 8/10 | - |
| [Ochi et al., 2015] | (1) Whole-body vibration device  (2) Horizontal leaning cable; EMG | (1) Performance of physical exercises w/ and w/o body vibration  (2) Simulated forward fall | EMG | healthy | n=20 | age: 65+  M/F: 0/20 | 5 months |
